# Dental hygienists’ perceptions of professionalism are multidimensional and context-dependent: a qualitative study in Japan

**DOI:** 10.1186/s12909-017-1107-9

**Published:** 2017-12-29

**Authors:** Yukiko Nagatani, Rintaro Imafuku, Toshinobu Takemoto, Tadayuki Waki, Taiji Obayashi, Tetsuji Ogawa

**Affiliations:** 1 Hiroshima University Hospital, 1-2-3 Kasumi Minami, Hiroshima, Hiroshima 734-8551 Japan; 2 Gifu University, Medical Education Development Center, 1-1 Yanagido Gifu, Gifu, 501-1194 Japan; 3 Hiroshima University, Graduate School of Biomedical & Health Sciences, 1-2-3 Kasumi Minami, Hiroshima, Hiroshima 734-8553 Japan; 4 Fukuyama University, Department of Human Culture, 1 Sanzo, Gakuen-cho, Fukuyama, Hiroshima, 729-0292 Japan; 5 Hiroshima University Hospital, Advanced General Dentistry, 1-2-3 Kasumi Minami, Hiroshima, Hiroshima 734-8551 Japan; 6 Hiroshima University, Graduate School of Letters, 1-2-3 Kagamiyama, Higashi-Hiroshima, Hiroshima 739-8522 Japan

**Keywords:** Professionalism, Dental hygienist, Super-aging society, Competencies, Dental hygiene education in Japan

## Abstract

**Background:**

Due to the declining birth rate and aging of Japanese society, the roles and responsibilities of dental hygienists are continuously expanding. Medical professionalism needs to be pursued continuously throughout one’s career in order to improve dental care and treatment. Although conceptualising professionalism is essential to the education of health professionals, professionalism in the field of dental hygiene has not been defined or adequately examined in Japan. The purposes of this study are to investigate dental hygienists’ perceptions of the constituent elements of professionalism and the factors affecting their perceptions.

**Methods:**

Semi-structured interviews were conducted with 18 dental hygienists in Japan. Drawing on the conceptualisation of professionalism in medicine described by Van de Camp et al., the transcribed data were thematically analysed.

**Results:**

The dental hygienists in this study perceived 70 constituent elements that were categorised into eight core competencies related to professionalism. These competencies were further classified into three main themes: intrapersonal, interpersonal, and public professionalism. There were three sociohistorical factors that affected their perceptions of the constituent elements, namely academic background (university or technical school), the contexts of any previously provided dental care (university hospital or dental clinic), and their social interactions with their colleagues during their engagement in dental practice (dental team or interprofessional team). Moreover, according to their sociohistorical backgrounds, the dental hygienists saw themselves variously as scholars (university graduates), facilitators (university hospital), skillful artisans (dental clinic), or collaborators (interprofessional team).

**Conclusions:**

Dental hygienists’ perceptions of professionalism are multidimensional and context-dependent, so culture- and professional-specific elements need to be included in educational curricula and continuing professional development programmes. In particular, the conceptualisation of professionalism in the field of dental hygiene as described in this study can be a springboard for enhancing undergraduate education and clinical training.

**Electronic supplementary material:**

The online version of this article (10.1186/s12909-017-1107-9) contains supplementary material, which is available to authorized users.

## Background

### Professionalism

Professionalism is defined as the moral understanding among a group of professionals that gives concrete reality to the profession’s social contract with the public [1]. Since the ancient Greek era, ‘the social contract’ has been considered a pledge by means of which a professional group dedicates itself to the enhancement of the public interest in the group’s specialty field. In return for meeting their social commitments, the professionals, such as medical doctors, priests, educators, lawyers, or engineering technicians, were originally granted a special status and exclusive rights in their practice. Since then, professionalism has been regarded as an important learning outcome in various professional education programmes [2–4].

Medical professionalism needs to be pursued continuously throughout an individual’s professional career in order to improve patient care and treatment. However, professionalism was not clearly defined as an element of competence for a long time. Rather, professionalism was a vague concept related to behavioural and cultural norms and clinical ethics. Since ‘the new millennium’ was issued in 2002, professionalism has been gradually recognised as an essential element in becoming a physician [5–7]. For instance, Stern provides a clearer model that classifies the constituents of professionalism into six values (i.e. responsibility, respect, competence, service-mindedness, integrity, and fairness) [5]. By contrast, some studies argue that professionalism is itself one of the competencies [8]. Thus, although competency and professionalism are related, both terms are used interchangeably, adding to the confusion surrounding their definitions [9].

This study regards professionalism as a broader term, including a combination of competencies, behaviour, and ethics [5, 9]. In other words, professionalism in this study refers to ‘the ability to demonstrate, through knowledge and behaviour, a commitment to the highest standards of competence, ethics, integrity, responsibility, and accountability in all professional endeavours’ [10]. Competency in this study is defined as ‘the ability to perform tasks at a certain skill level’, which is listed as one element of professionalism [9].

### Professionalism of dental hygienists in different cultural contexts

Although the nature of professionalism has been discussed in different countries and regions over time, the educational stances toward professionalism vary across professional fields, cultural and historical backgrounds, and healthcare systems [11]. This is because the expected (or regulated) roles and responsibilities of the dental hygienist (DH) are different across countries. For instance, in Australia, Canada, and the U.S., DHs have developed their professional autonomy, and their clinical roles include a variety of duties, such as performing overall oral health assessments, taking and developing dental radiographs, administering local anaesthetic injections, and sculpting materials to fill cavities. Furthermore, in order to advance the profession of DH, there are continuous discussions on what new initiatives DHs can take for patient-centred care, such as research activities [12]. In this context, professionalism, including core competencies for DHs, has been well-defined. For instance, in Canada, the concept of professionalism has been developed to encompass a range of abilities required of all DHs, such as responsibility, accountability, knowledge application, continuing competence, professional relationships, and the DH–client relationship [13]. In the U.S., the professional roles of the DH are classified into clinician, corporate, public health, researcher, educator, administrator, and entrepreneur [14]. In recent years, an evidence-based approach to oral care, problem-solving skills, communication, and interprofessional collaboration have been considered as essential skills for DHs worldwide [15, 16].

Learning professionalism has to be developed in accordance with the social needs and cultural contexts of the profession [11, 17]. In Western countries, dental hygiene courses and learning experiences have been created throughout the curriculum with the intent of teaching professionalism, including a professional responsibility thread. These courses cover the patient–provider relationship, ethics, academic service learning, and leadership and professional development [18]. To improve the effectiveness of the teaching of professionalism, some educational institutions have integrated mindfulness practice into the dental hygiene curriculum [19].

In contrast to the DHs in the above-mentioned countries, the fundamental roles and responsibilities of DHs in Japan are limited to dental prophylaxis, dental assistance and dental health education. However, the roles of DHs are now expanding in a contemporary Japanese society that is rapidly changing due to its declining birth rate and the aging of its population. This situation has caused some major changes in the social and disease structures. In particular, DHs are increasingly expected to contribute to the enhancement of patient quality of life and to support patients’ family members through interprofessional collaboration, in addition to the three ‘original’ roles of DHs [20]. To meet such societal needs, DHs are required to be further engaged in continuing their professional development through reflective practice on their experiences [15].

The structure of dental hygiene education in Japan has been reformed with the expansion of the roles and responsibilities of DHs. Specifically, education originally consisted of a one-year programme in 1949 (and continued until 1983), but a two-year programme was introduced in 1958 (and continued until 2010). In 2005, a three-year programme was adopted, which was fully replaced by a two-year programme in 2010. Moreover, a four-year undergraduate programme was initiated in 2004 [21]. (Differences in the educational purposes and expectations of the different programmes are shown in a Additional file 1). Consequently, contemporary oral care in Japan involves DHs with different educational backgrounds.

In the transition to the three-year programme, Shimokawabe emphasised humanity, clinical knowledge and skills, research, and internationality as the learning outcomes of an undergraduate dental hygiene education [22]. To date, however, the professionalism of DHs in Japan has not been adequately defined and examined. In contrast to Western countries, professionalism has not been systematically incorporated into contemporary training programmes for DHs in Japan [23]. In other words, we are developing the concept of professionalism and its application to the training of DHs.

### Aims of the study

Lacking professionalism guidelines for DHs in Japan, we conducted a pilot study showing that a DH’s age and experience partially characterise their perceptions of professionalism [24]. Specifically, the scientific aspects, including clinical knowledge and skills, are mainly acquired when a DH is in their 20s; interpersonal qualities, such as honesty and integrity, develop in their 30s; and social aspects, such as the profession’s social contract, future prospects, and additional training, develop in their 40s.

A better understanding of how DHs with different educational backgrounds acquire and develop their conceptualisations related to professionalism would allow us to enhance contemporary undergraduate and postgraduate education in Japan. Moreover, the findings from a study performed in a super-aging society in which more than 21% of the population is 65 years or older [25], such as Japan, will also provide insight into health professional education and DH education in other countries. The purpose of this study was to define and develop an educational model of professionalism for DHs. In order to achieve this, we formulated the following research questions:

1. What constituent elements of professionalism are perceived by DHs in Japan?
2. What factors influence their perceptions of the constituent elements of professionalism?

## Methods

### Participants

The participants in this study were DHs in Japan who were certified by the Ministry of Health and Welfare. The number of DHs in Japan exceeded 100,000 in 2010. Over 90% of DHs work in dental clinics and around 5% are involved in dental care in hospitals [26]. DHs are expected to participate in oral health promotion and preventive care. Specifically, three main roles of DHs have been clarified: preventing dental caries and periodontitis, assisting dentists in dental practice, and providing patients with instructions on how to implement proper dental care as a way to enhance quality of life.

We randomly selected four out of the 27 hospitals that participated in our pilot study. We then asked the directors of the oral health divisions in these healthcare institutions to recommend 40 DHs who were actively involved in clinical practice, research and education activities. Using purposive sampling, we selected 18 DHs, who were currently working at university hospitals, using the criteria of age, clinical experience, and academic background. Table 1 provides details regarding these research participants.

**Table 1:** Research participants

DH	Age	Educational degree	Type of institution^a^	Years of active practice	Previous practice setting	
					General practice in dental clinic	Hospital practice or nursing home with IPW
A	20s	Bachelor Master	Undergrad. Postgrad.	1		
B	20s	Certificate	Technical college	8	✓	
C	20s	Diploma Master	College Postgrad.	3		
D	20s	Bachelor Master	Undergrad. Postgrad.	3		
E	20s	Bachelor	Undergrad.	5	✓	
F	30s	Diploma	Collage	18		
G	30s	Diploma	College	16		
H	30s	Certificate	Technical college	19	✓	
I	30s	Diploma	College	9	✓	
J	30s	Diploma	College	16	✓	✓
K	40s	Certificate	Technical college	17	✓	✓
L	40s	Certificate	Technical college	20	✓	
M	40s	Diploma	College	23		
N	40s	Certificate	Technical college	27	✓	✓
O	50s	Certificate	Technical college	37		
P	50s	Certificate	Technical college	35		
Q	50s	Certificate	Technical college	32		
R	50s	Certificate	Technical college	21	✓	✓

^a^Postgraduate school (two-year), Undergraduate school (four-year), College (two- or three-year), Technical college (two-year)

### Data collection

Data were collected through semi-structured interviews that were conducted in Japanese and lasted between 40 and 90 min, approximately. To ensure a safe environment that would elicit the interviewees’ straightforward beliefs and experiences as DHs, the interviews were conducted in a small conference room in their workplace. All the interviews were performed by the first author, YN, who is a DH at a university hospital and has received formal training in interviewing as a qualitative research method in her doctoral study.

In the semi-structured interviews, we posed two core questions about 1) the interviewees’ perceptions of an ideal DH as a health professional and 2) the necessary attitudes, skills, and abilities required by DHs. In addition to these core questions, we elicited background information about 4) the interviewees’ overall journey to becoming a DH, 5) their reasons for becoming a DH, 6) the gaps between the reality of working at the oral healthcare site and what they learned in undergraduate education, and 7) their motivation to engage continuously in oral care as a DH. The interviewer generally followed the guide, but was able to follow topic trajectories in the conversation. During the conversation, using probing questions, she gathered as much information related to the study aims as possible.

The audio-recorded data of interviews were transcribed by the authors. We asked a professional service company to translate Japanese transcripts into English, and the second author, RI, checked the accuracy of the translation in terms of the nuances of speakers’ meaning. This study was approved by the Institutional Review Boards at Hiroshima University (494–3) and Gifu University (26–244).

### Data analysis

To interpret the interview data, this study drew on ‘themes within professionalism and associated elements’ as classified by Van de Camp et al. [27]. Their study found three themes within the concept of professionalism: interpersonal professionalism, public professionalism, and intrapersonal professionalism. These pre-identified themes were simultaneously examined with a more inductive labelling process for qualitative data [28].

The thematic analysis method used in this study involved generative coding and theorisation. First, the text data were divided into small units and classified as meanings, actions, events or ideas. Second, each of these small units was labelled with an interpretive description, and these labels were then consolidated into professionalism-related main categories as the core elements for becoming a DH. At this stage, the study drew on the findings of the previous study to interpret the qualitative data [27]. Lastly, by comparing the categories using a contrastive procedure, the unique character of each category and its relationship to the other categories were described to develop a story line.

To enhance the trustworthiness of the qualitative analysis, three researchers (YN, RI, and TeO) were independently involved in coding and categorising the data, before cross-checking their data interpretation and analysis. The preliminary findings of the analysis were carefully reviewed multiple times by all the members of the research team, including TW, TT and TaO, to establish the validity of the data analysis. Theoretical saturation was achieved on the basis of the authors’ agreement after analysing the interview data of the 19th participant.

## Results

### Constituent elements of professionalism for DHs

In total, this study identified 70 constituent elements related to the concept of professionalism as perceived by DHs in Japan. These constituent elements were categorised into eight core elements, which were then classified into three main themes: intrapersonal, interpersonal, and public professionalism (see Table 2).

**Table 2:** Constituent elements of professionalism perceived by Japanese DHs

Intrapersonal professionalism	
Personality	Faith, honesty, earnestness, passion, affection, virtue
Ethical behaviour	Justice, morality, fairness, altruism, autonomy
Lifelong learning	Pursuit of expertise, reflective practice, inquiring mind, self-improvement, spirit of progress, curiosity
Interpersonal professionalism	
Caring for patients	Facing, learning from each other, faith in life’s meaning and value, being thoughtful, compromising, understanding others’ point of view, acceptance of self, acceptance of others, empathy, compassion, consideration
Interprofessional collaboration	Dissemination ability, flexibility, being influential, multifaceted vision, trust, rapport building, adaptation to circumstances, comprehensive thinking, viewpoint of primary care, management, mutual understanding among professionals, cooperativeness, problem presentation, internationalism
Public professionalism	
High level of expertise	Knowledge, clinical skills, evidence-based medicine, educational ability, logical thinking, ability to find and solve problems, reflection, Inference-making/reasoning abilities, good clinical judgement, being competent person, working efficiently
Roles and responsibilities	Contribution, obligation, self-consciousness, sense of mission, being a role model, career development, sense of responsibility
Vision for societal and organisational development	Independence, skills to overcome adversity, innovative ability, creativity, ability to respond at work, autonomy of professional associations, being active, planning ahead, leadership, clarification of professional values

According to Van de Camp et al. [27], intrapersonal professionalism covers ‘demands that have to be met to function effectively and adequately in the medical profession as an individual’. Intrapersonal professionalism involves three core elements: personality, ethical behaviour, and lifelong learning. Interpersonal professionalism was defined as the ‘elements of professionalism that refer to prerequisites for effective and adequate contact with patients and other healthcare professionals’. Interpersonal professionalism encompasses two core elements: caring for patients and interprofessional collaboration. Public professionalism covers the ‘elements of professionalism that relate to the demands society places on the medical profession’. Public professionalism includes three core elements: high level of expertise, roles and responsibilities, and vision for societal and organisational development.

### Factors that influence perceptions of professionalism

This study found that the interviewees’ sociohistorical backgrounds characterises their perceptions of DH professionalism. The first factor is their academic background. Specifically, there are different characteristics of perceived professionalism between participants who graduated from a university (or postgraduate school) or a technical college. This study found that the university graduates focused more on public professionalism. They especially emphasised their ability to determine and solve a problem and find a sense of mission:

*‘You should always maintain a positive learning attitude. Don’t deal with familiar jobs just routinely every day. Many dental hygienists around me just leave their questions unsolved or tasks unfinished. You shouldn’t be like that’. —Participant E.*

*‘I think the benefit of being a hygienist at the hospital is the opportunity to be involved in oral care on a ward. However, I feel it’s a pity that the hospital can’t effectively use the ability of dental hygienists. I’m sure there are many patients who would like to improve their oral health, which is related to the condition of the whole body. We could alleviate, for example, postoperative pain as much as possible, but we don’t have the opportunity to serve those patients. Our university rarely offers that to us, so we have to do something in the current situation’. —Participant C.*

Likewise, the technical college graduates prioritised public professionalism. However, most of their comments were related to clinical skills, such as skills in providing dental treatment and care:

*‘In general, dental hygienists need to improve their clinical skills at first. After graduation from the school, I focused on developing my skill as a dental hygienist. If I did not have this experience, my view of dental treatment and care might be limited’. —Participant H.*

The second factor is the setting of any previously provided dental care (i.e. at a dental clinic or university hospital). The participants who had clinical experience at a dental clinic tended to value inference-making and reasoning abilities which are categorised as skills that require a high level of expertise:

*‘For a dental practitioner, it’s important to rotate the number of patients efficiently. I understand that he has to take care of the reception over there and grasp the situation here (in the treatment room) at the same time, and that usually makes a private practitioner busy. Consequently, I was always thinking things like “I could knead the impression material after finishing this” or “I think there was still some plaster left…”. Because it was all in a day’s work, I was always on the lookout for things happening around me’. —Participant H.*

By contrast, the participants who had worked continuously only at a university hospital emphasised the importance of evidence-based medicine in public professionalism and interpersonal professionalism among colleagues:

*‘The university is at the top, you know, so the DHs there perform proper hygiene management at a high level. I think their outstanding performance is achieved on the basis of evidence from clinical studies. That is, evidence-based practice is really important to the DHs’. —Participant O.*

*‘I often see people cooperating with each other. It’s probably because I work at the hospital. I think it’s very important to have a connection with people such as doctors and other various staff members, as well as the ability to communicate with people, including not only patients but also the staff members around you, as human beings’. —Participant D.*

The third factor is the DH’s social interactions with other colleagues while engaging in one’s current (or previous) dental practice (i.e. interprofessional team or dental team). The participants who had been involved in interprofessional care emphasised the importance of ‘faith in life’s meaning and value’ and ‘compromise’ in terms of interpersonal professionalism elements:

*‘Because it was a residential home, literally anything could happen in the special nursing home for the elderly where I worked before. It may be too much to call it ‘the way of life’ of the person, but it’s a place where you can see at least how the elderly person is living his own life. So, actually, it’s a place where you have to respect his life’. —Participant N.*

*‘It’s important to clean (the oral cavity), but just cleaning is not enough. I think it’s important that it suits the situation and condition of the patient. His teeth may be still stained afterwards, but if they look the best for him at that time, and if we have done something for him, I guess it will be ok. You don’t have to perform perfect cleaning. That’s not necessary. I developed that sense in the special nursing home for the elderly’. —Participant N.*

On the other hand, the DHs who were a part of a dental team were more concerned with the ‘pursuit of expertise’ in public professionalism and ‘contributing’ in intrapersonal professionalism:

*‘I will be happy if I can find an interesting (dental) field for me. Once I get intrigued by something, I will investigate it thoroughly’. —Participant G.*

*‘The skill I have is (a willingness) to serve patients, which I believe only hygienists can do. That’s all the competency I have, and that’s all I can do. That’s why I am a hygienist. Probably, I just want to be of service to someone. That’s why I can’t quit my job as a hygienist. In other words, I can only be a hygienist’. —Participant J.*

## Discussion

### Characteristics of perceived professionalism by DHs from different backgrounds

This study illustrates Japanese DHs’ perceptions of professionalism and the factors that influence their perceptions. The findings in this study corroborate a previous study that indicated that the concept of professionalism is multidimensional [27]. Specifically, the DHs in this study perceived the constituent elements of professionalism differently, in accordance with their sociohistorical backgrounds. As Table 3 shows, four main groups related to the professionalism of DHs in Japan were identified.

**Table 3:**
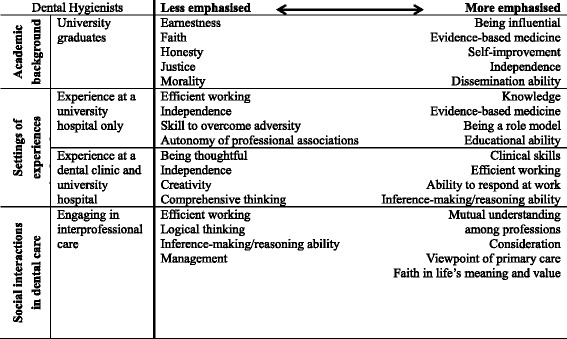
Characteristics of perceived professionalism by DHs with different backgrounds

First, DHs who received a university education tended to see themselves as scholars who could advance expertise by practicing oral care. They focused more on self-improvement, exercising influence, and their ability to disseminate. However, it has to be noted that all DHs in this study who graduated from a university or postgraduate school were less than 30 years old. Further studies are needed to explore how one’s educational background influences perceptions of professionalism.

Second, DHs who had worked only at a university hospital tended to see themselves as facilitators who could effectively support patients and educate DHs at entry-to-practice. They stressed being a role model and advancing their expertise by using an evidence-based approach to oral care. Their perceptions were derived from the clinical and educational culture of the university hospital, which stresses the importance of providing dental care in a scientific manner based on objective clinical data. On the other hand, there were few comments on efficient working, the autonomy of professional associations, independence, and skills to overcome adversity in this group. This is because the DHs tended to see themselves as followers of their dentists and believed that independence as health professionals was not necessarily pivotal to the role of DHs at a university hospital in Japan.

Third, DHs who had experience of a dental clinic tended to see themselves as skillful artisans who take an experiential approach to patient oral care. They valued their better understanding of a ‘situation’ in order to provide treatment and care to patients efficiently. For instance, they made more comments about clinical skills, inference-making ability, and efficient working. On the other hand, they did not mention interprofessional collaboration as an element of professionalism because they rarely worked with other health professionals in the dental clinic. Furthermore, there were no comments on caring for patients, including comments regarding empathy, consideration and compassion.

Fourth, DHs who had been involved in interprofessional care tended to see themselves as collaborators who supported their patients and family members from the perspective of providing primary care. They prioritised interpersonal professionalism, including caring for patients and collaborating with other professionals. They had rich experiences in providing oral health care interpersonally. For instance, they had dealt with the deaths of patients in holistic care and had provided family support, in addition to providing care during home visits in collaboration with other professionals.

Although the perspectives in emphasising the elements of professionalism varied according to the educational backgrounds and clinical experiences of the DHs, this study provides an overall framework of how professionalism is perceived among DHs in Japan. The responsibilities and roles of DHs in Japan need to be defined continuously in order to meet the demands of a super-aging society. Whereas DHs were previously allowed to be involved in dental practice while under the instruction of dentists, under the amendment of the Dental Hygienist Act (which was passed in Japan in 2015), DHs are now expected to collaborate with physicians and dentists in order to enhance patient oral health. Therefore, as the social expectations regarding DHs change, they need to participate more independently in patient care with a firm sense of mission in order to innovate and overcome adversity. Furthermore, in a super-aging society, DHs are increasingly expected to contribute to interprofessional care and the tenets of interpersonal professionalism will become more important to them, including ‘comprehensive thinking’, ‘leadership’, ‘being influential’ and ‘cooperativeness’ (see Table 2).

### Professionalism across professions and countries

The constituent elements of professionalism are differently perceived and valued across professions. Nishigori et al. suggested that the professionalism of Japanese physicians corresponds to the concept of *bushido* and its seven principal virtues: rectitude (*gi*), honesty (*sei*), benevolence (*jin*), politeness (*rei*), courage (*yu*), honour (*meiyo*), and loyalty (*chugi*) [17]. In *bushido*, these elements are generally explained as a code of conduct for society [29] . Furthermore, Van de Camp et al. found that, in comparison with DHs, societal development and ethical behaviour were emphasised more among medical professional, including accountability, the social contract, compliance, and confidentiality [27]. Because physicians have more opportunities to be directly involved in patient life as a part of their practice, their professionalism can be closely connected with their social roles and responsibilities.

Professionalism in nursing is prescribed with a greater focus on caring for patients, self-management, and self-improvement [30, 31]. Because nurses spend relatively more time with patients, they have more opportunities to confront critical situations in healthcare than do DHs. Thus, in the given context of nursing, nurses are required to perform self-management and self-evaluation as part of their nursing practice, which is pivotal to providing holistic patient care.

In addition to the professionalism demonstrated by different professions, the constituent elements are also perceived differently across countries [13, 32, 33]. For instance, DHs in the U.S. value leadership in a medical team, responsibility for sharing information, and the planning of effective advance care [10, 33]. DHs in the U.S. have established the social status and autonomy of a health profession to a greater extent than have DHs in Japan. In other words, the different social expectations regarding DHs may result in different perceptions and values regarding the professionalism of the field of dental hygiene.

As Al-Rumayyan et al. suggested [29], professionalism is contextually dependent, so culture- and professional-specific elements need to be included in educational curricula and continuing professional development programmes. In defining professionalism for DHs, their social expectations, career phases, and sociohistorical backgrounds need to be taken into account. In particular, disease, population, and sociopolitical structures differ across communities and countries. The expectations of each profession also differ in societies. Thus, the findings of this study suggest that professionalism is a multidimensional and context-dependent concept.

### Implications for practice

The findings imply that when developing education programmes in countries that have not yet defined professionalism for DHs, the Western framework of professionalism cannot be ‘imported’ and ‘adopted’ straightforwardly, but can be applied to develop a culture-tailored framework for medical professionalism. In other words, we need to note that there is no single framework of professionalism that fits all educational contexts [29].

This study has revealed that DHs’ academic backgrounds, clinical experiences, and social interactions in healthcare shape their perceptions of professionalism, which is a longitudinal process of becoming a professional. This finding suggests that when incorporating professionalism into the training of DHs, we need to direct attention to the processes by which their professional identity, humanistic values and behaviour are cultivated [34]. In this regard, Cruess, Cruess and Steinert [35] argue that a fifth level ‘Is’ should be added at the apex of Miller’s pyramid (i.e, knows, knows how, shows how, and does) in the movement to ensure that professionalism is taught throughout the continuum of medical education. ‘Is’ refers to the being or identity related to who we are and who we want to become. Furthermore, Irby and Hamstra [36] have highlighted identity formation as a key perspective in cultivating professionalism, and we need to distinguish between professionalism as a trait (character or behaviour) and professionalisation as a process (identity formation and development).

Methodologically, this study highlighted the strengths of a qualitative research approach to exploring the perceptions of professionalism. Specifically, it allowed the researchers to provide a rich narrative description of the multidimensional and context-dependent nature of professionalism perceived by DHs in Japan, which cannot be investigated from a psychometric analytical perspective. However, the findings are not generalisable due to the small number of participants. Further qualitative studies need to be conducted to include other DHs, such as DHs who are currently working in a dental clinic.

## Conclusions

The purpose of this study was to specify the constituent elements of professionalism perceived by DHs in Japan. Eight core elements of professionalism emerged, and they were grouped according to three themes: intrapersonal professionalism (including personality, ethical behaviour and lifelong learning), interpersonal professionalism (including caring for patients and interprofessional collaboration), and public professionalism (including high level of expertise, roles and responsibilities, and vision for societal and organisational development). In addition, there were three sociohistorical factors that affected DHs’ perceptions of the constituent elements: their academic background (i.e. university or vocational school), the contexts of any previously provided dental care (i.e. university hospital or dental clinic), and their social interactions with other colleagues in their engagement of dental practice (i.e. dental team or interprofessional team). This study sheds light on conceptualising professionalism in health professions in a super-aging society. This conceptualisation, based on Van de Camp et al.’s framework [27], can be a springboard for enhancing undergraduate education and clinical training in relation to professionalism.

## Additional file

Additional file 1: Different outcomes and expectations of the different programmes. (DOCX 17 kb)

## Abbreviation

DH: Dental hygienist
