# Supplementary material for: Dental hygienists’ perceptions of professionalism are multidimensional and context-dependent: a qualitative study in Japan
Source: BMC Med Educ. 2017 Dec 29;17:267. doi: 10.1186/s12909-017-1107-9 (PMC5747186; doi:10.1186/s12909-017-1107-9)
Supplement: Additional file 1: — Different outcomes and expectations of the different programmes. (DOCX 17 kb) [file 12909_2017_1107_MOESM1_ESM.docx]

| Programme | Learning outcomes and expectations | | | |
| --- | --- | --- | --- | --- |
| One-year  (1949-1983) | Prevention of dental disease |  |  |  |
| Two-year  (1958-2010) | ✓ | Dental assistant  Oral health instruction to patients |  |  |
| Three-year  (2005-present) | ✓ | ✓ | Public health  Home visit care |  |
| Four-year  (2004-present) | ✓ | ✓ | ✓ | Community-based oral care  Caring for elderly  Social welfare  Interprofessional care  Clinical research |

Additional file 1: Different outcomes and expectations of the different programmes
